# A response to the Comment on Tomaskova et al. Mortality in Miners with Coal-Workers’ Pneumoconiosis in the Czech Republic in the Period 1992–2013. *Int. J. Environ. Res. Public Health*, 2017, *14*, 269 by the Author Mei Yong

**DOI:** 10.3390/ijerph15020322

**Published:** 2018-02-13

**Authors:** Hana Tomášková, Anna Šplíchalová, Hana Šlachtová, Daniela Pelclová, Pavel Urban, Zdeněk Jirák

**Affiliations:** 1 Institute of Public Health, 70200 Ostrava, Czech Republic; anna.splichalova@zuova.cz (A.S.); hana.slachtova.x@gmail.com (H.S.); zdenek.jirak@osu.cz (Z.J.); 2 Department of Epidemiology and Public Health, Faculty of Medicine, University of Ostrava, 70103 Ostrava, Czech Republic; 3 Department of Occupational Medicine, 1st Faculty of Medicine, Charles University in Prague, 12108 Prague, Czech Republic; daniela.pelclova@LF1.cuni.cz (D.P.); pavel.urban@szu.cz (P.U.); 4 The National Institute of Public Health, 10042 Prague, Czech Republic

At first, we would like to thank Mei Yong for the comments on our article Mortality in Miners with Coal-Workers’ Pneumoconiosis in the Czech Republic in the Period 1992–2013 in *Int. J. Environ. Res. Public Health* [1]. We agree that answering the question of causality of lung cancer in coal-workers’ pneumoconiosis (CWP) is of crucial importance. However, the aim of our work was not to answer this key question, but based on our data, we aimed to evaluate the total and specific mortality of black coal miners in comparison with the mortality of the unexposed population of the same age in the Czech Republic (CR).

## 1. The Comment on the Goal of the Work, Estimation of the Dust Exposure and the Different Age Composition

First of all, it should be mentioned that most of Mei Yong’s comments were discussed by the authors Tomášková et al. in both the Introduction and Discussion sections of the above paper. In this study, two cohorts of miners were analysed. The first cohort was comprised of the miners who did not fall ill with CWP and were preventively replaced from the work in the underground after reaching 100% of the maximum permissible exposure (MPE), and the other of the miners who suffered CWP. The authors studied whether the mortality of the sample of preventively-replaced miners who did not fall ill with CWP or only its light form was comparable with the common population of the CR. An exact description of how the MPE was determined was provided in the beginning of the paper (the possible previous exposure from other mine workplaces was included in the MPE) as well as the history of its implementation in the CR. The dust exposure of miners included in the sample without CWP was well defined by the total dose of inhaled dust and quartz. Since the preventive replacement of miners had been gradually applied since 1986 and it came fully into force in 1991, the sample contained the miners without CWP and a portion of miners whose 100% MPE was slightly exceeded in the time of replacement. MPE in drifters and coal extraction was reached on average after about 3500 shifts (i.e., after about 17.5 years of the work underground). In high-risk workplaces where the permissible limit of dust was exceeded several-fold, the MPE could be reached after 10 years. The average age of the cohort without CWP in the beginning of the follow-up was 44.0 ± 6.3 years. The second cohort comprised the miners with diagnosed CWP (the doubt diagnostics of CWP will be discussed later). These miners reached the highest permissible dose of dust (complying with the MPE) before the year 1991, and were replaced on the bases of an acknowledged form of CWP within the preventive examinations or they were miners with the diagnosed light form of CWP before they reached the MPE. As confirmed in the control study, this was less than 5% of miners from the total number of miners who reached 100% MPE [2]. The individual data on dust exposure in the sample of miners with CWP was not available in this study because the National Register of Occupational Diseases does not contain this data. The estimation of the dust exposure in the individual CWP categories could be provided according the equations expressing the relationship between the level of retention and a cumulative dose of dust and quartz on one hand and between a morphological finding and a radiographic finding on the other hand. The equations were published in an earlier work [3] and were obtained based on the analysis of necrotic material of the lungs of 111 miners who died in the period from 1980 to 1990.

Radiographic lung findings in this group were available from the start of work in mining to a miner’s death. The findings were evaluated by a group of three experts according to ILO international classification of radiographs of pneumoconiosis (1980). The average cumulative dose of respirable fraction SiO_2_ and corresponding retention of quartz in lungs for the initial CWP form was estimated at 8.59 g (retention 0.331 g), for the simple CWP at 12.69–16.34 g (retention 0.449–0.567 g) and for the most severe form—CWP with the deposit type C, at 20.88–29.08 g (retention 0.685–0.921 g). In the cohort of miners with CWP, miners with the simple CWP (71%) prevailed, which implies that they had to have reached the MPE before implementation of the preventive measures. This corresponded with a higher average age at entry into the study (49.70 ± 12.37 years). Miners with CWP were therefore on average 5 years older compared with the cohort without CWP.

## 2. The Use of the Standardized Mortality Ratio (SMR) for Evaluation

It is necessary to point out that the cohorts were not compared between each other, but the goal was to compare the cohorts with the unexposed Czech population with the use of SMR—a standard tool used for age adjustment. In the case of deaths for respiratory diseases in the cohort of miners without CWP (61.1 years (SD = 8.5) vs. 70.5 years (SD = 10.9) in miners with CWP), it was necessary to take particular account of age at the beginning of the study as well as the proportion of miners who died of this disease from the total number of deaths (without CWP—6%, with CWP—14%).

## 3. The Questioned Diagnoses of CWP

Please allow us to clarify the issue of CWP diagnosis. In the CR, the radiographic examination of miners is carried out in the first years of exposure in two-year and then one-year intervals. The evaluation is based on a big back-front radiographic lung finding, and is provided exclusively by physicians from the specialized workplaces—clinics (departments) of occupational diseases. The evaluation is performed by the head physician of the department (clinic), usually with the presence of a specialist in pneumology and another physician (physicians) depending on the workplace size, using the international radiographs ILO 1980 classification. The results are sent into the National Register of the Occupational Diseases together with notifying signs according ILO 1980 [4].

## 4. The Lack of Data on Smoking and Statistical Methods

The lack of data on smoking is a limitation of the study, as was already acknowledged in the discussion. A great effort has been focused on the completion of data on smoking, and the data collection is ongoing. However, as discussed in the paper, the issue of smoking is not related only with the presence/absence of this data, but also with the timing of when this data was obtained. Due to the comparison of smoking status between the cohorts without and with CWP, it can be assumed that miners stopped smoking in the event of health disorders, as we mentioned in the paper. The data in miners without CWP was found at the time of replacement, and in miners with CWP this data was completed from the medical records. Based on this finding, it would be appropriate to have available detailed information on the time and intensity of smoking for the analysis of the impact of smoking and CWP diagnosis on the development of lung cancer. The authors are aware of the need to use recommended statistical methods; unfortunately, it was not feasible to use them based on the data available in this study.

In the Discussion section of the paper, other possible factors related to lung cancer are introduced—moulds and ionizing radiation.

## 5. The Questioned Causes of Deaths in the Case of Lung Cancer

Mei Yong also doubted the determination of the cause of death for lung cancer. This problem was also discussed in the paper, and the authors stated how they would deal with the problem. In our case, in the CR, the compulsory reporting of oncological cases has been implemented since the year 1953 [5], and based on this register we can compare the data on incidence and mortality for lung cancer. This comparison has already been finished, and is in preparation for publication. Based on our preliminary data, we can confirm that the above bias did not appear in miners with CWP in the CR.

## 6. Conclusions

The main objective of the study was to compare the mortality of miners with and without CWP and the age-corresponding population of men in the CR. The goal of the preventive measures was to avoid the exposure leading to the development not only of the light but especially the severe form of this disease. This fact was verified [2]. The results of the presented study show that the total mortality in miners replaced based on the MPE (without CWP or with the light form of CWP) is not higher than in the general population. In our study, the mortality was even lower that can be ascribed to the “healthy worker” effect. In addition, the study showed that if miners fall ill with the severe form of CWP or CWP in relation with TBC, the total mortality was significantly higher compared with the general population. The question arises as to how mortality in the cohort of miners without CWP will develop.

Further studies are also needed to answer questions raised by Mei Yong.
